# Comparison of usability evaluation methods for a health information system: heuristic evaluation versus cognitive walkthrough method

**DOI:** 10.1186/s12911-022-01905-7

**Published:** 2022-06-18

**Authors:** Mehrdad Farzandipour, Ehsan Nabovati, Monireh Sadeqi Jabali

**Affiliations:** 1 Health Information Management Research Center, Kashan University of Medical Sciences, Kashan, Islamic Republic of Iran; 2 Department of Health Information Management and Technology, Kashan University of Medical Sciences, Kashan, Islamic Republic of Iran

**Keywords:** Hospital information system, Heuristic evaluation, Cognitive walkthrough, User-computer interface

## Abstract

**Background:**

There are differences of opinion regarding the selection of the most practical usability evaluation method among different methods. The present study aimed to compare two expert-based evaluation methods in order to assess a nursing module as the most widely used module of a Hospital Information System (HIS).

**Methods:**

Five independent evaluators used the Heuristic Evaluation (HE) and Cognitive Walkthrough (CW) methods to evaluate the nursing module of Shafa HIS. In this regard, the number and severity of the recognized problems according to the usability attributes were compared using two evaluation methods.

**Results:**

The HE and CW evaluation methods resulted in the identification of 104 and 24 unique problems, respectively, of which 33.3% of recognized problems in the CW evaluation method overlapped with the HE method. The average severity of the recognized problems was considered to be minor (2.34) in the HE method and major (2.77) in the CW evaluation method. There was a significant difference in terms of the total number and average severity of the recognized problems by these methods (*P* < 0.001). Based on the usability attribute, the HE method identified a larger number of problems concerning all usability attributes, and a significant difference was observed in terms of the number of recognized problems in both methods for all attributes except ‘memorability’. Also, there was a significant difference between the two methods based on the average severity of recognized problems only in terms of ‘learnability’.

**Conclusion:**

The HE method identified more problems with lower average severity while the CW was able to recognize fewer problems with higher average severity. Regarding the evaluation goal, the HE method was able to be used to improve the effectiveness and satisfaction of the HIS. Furthermore, the CW evaluation method is recommended to identify usability problems with the highest average severity, especially in terms of ‘learnability’.

**Supplementary Information:**

The online version contains supplementary material available at 10.1186/s12911-022-01905-7.

## Background

In recent years, healthcare organizations in many countries have primarily invested in developing and implementing various information technology systems [1, 2]. Moreover, the usability of health information technology systems has always been an area of concern for such organizations [1]. Well-designed health information systems can support the flow of clinical information in several ways, thereby improving patient care [3]. The nursing module of the Hospital Information System (HIS) can be regarded as one of the most crucial and widely used healthcare information systems [4], which needs to be integrated into nurses' daily practices [5]. In this regard, a system's efficient and effective use heavily depends on its proper design and the fulfillment of users' expectations and a poor design and improper usage can trigger several problems in accepting and using the system [6–8]. Problems would lead to user dissatisfaction [9, 10], increased error rates, and decreased safety and quality of patient care services [11]. Accordingly, various usability attributes should be concerned in developing interactive health information systems such as the nursing module [8]. According to the International Organization for Standardization (ISO), usability attributes encompass efficiency, effectiveness and satisfaction [12], while Nielsen considers learnability, efficiency, memorability, error and satisfaction as usability attributes of the information systems [13].

The usability evaluation of information system user interfaces can be performed by several methods [14]. These methods are divided into two groups: user-based testing methods, in which users identify the problems which prevent them from performing tasks, and expert-based inspection methods, in which experts identify general problems with the system user interface [15, 16]. Each of these methods has its own advantages [16].

The Heuristic Evaluation (HE) and Cognitive Walkthrough (CW) are two common expert-based evaluation methods, which can recognize system usability problems effortlessly, quickly and economically [15, 17, 18]. In the HE method, the system user interface is evaluated concerning a set of predefined principles, known as heuristics [19]. This method, performed by three to five evaluators, can recognize up to 80% of the usability problems [20]. As a task-specific and structured evaluation method, the CW evaluation method adopts the principles of cognitive psychology to simulate cognitive processes and user actions to perform specific tasks using a computer system [21, 22].

Although there are many usability problems in information systems, most problems can be recognized and modified by proper planning and selecting an appropriate evaluation method [23]. In other words, improving the usability of health information systems requires a deep understanding of various usability evaluation methods and relevant knowledge of how to implement these methods [1]. However, there might be several challenges and inconsistencies in selecting the most appropriate usability evaluation method and implementing the findings obtained from each method [23]. In this case, if there are a large number of evaluation methods available to be applied in a study, the findings of previous studies, particularly comparative studies, should be considered while selecting the most appropriate method so that they can be highly beneficial in describing the advantages and disadvantages of each method and predicting what is to be achieved by applying each method. Consequently, the most appropriate evaluation method can be selected based on the evaluation goal.

Numerous studies have compared the findings of the HE and CW evaluation methods and other usability evaluation methods [24–30]. In a study done by Khajouei et al. [28], the HE and CW evaluation methods were compared based not only on the number and severity of the recognized problems but also on a blend of ISO and Nielsen usability attributes. Khajouei et al.'s study [28], as the only research in this regard, was carried out on the clinical module of a medical office management system. Regarding the limitations of their concerned system, it was specifically designed for physicians with low functional capabilities and had a limited number of installations across the country compared to other health information systems. Accordingly, it seems necessary to perform similar studies to compare the abovementioned evaluation methods on large-scale health information systems with more capabilities, which are expected to have a more appropriate design due to the large number of their installations and users across the country. Also, the breadth of the evaluated information system makes the results of the comparison between the HE and CW methods more generalizable to other health information systems and we can be more confident about the results of comparing the two methods in the selection of the most appropriate evaluation method in other health information systems. This study aimed to compare the number, severity, and type of the recognized problems according to ISO and Nielsen’s usability attributes using the HE and CW evaluation methods in a nursing module of one of the largest HISs installed in many hospitals across the country.

## Methods

### System

The HE and CW evaluation methods were compared in the nursing module of Shafa HIS (developed by Tirajeh Rayaneh Co.). By the time this research study was conducted, the system had been installed in more than 200 hospitals across the country. It enjoys various capabilities as follows: admitting patients and allocating bed and room for patients in the inpatient ward, recording requests for paraclinical services and monitoring the results, recording requests for a surgical procedure, recording requests to transfer and move a patient to another ward and recording a patient's information at the discharge time. The nurses and secretaries working in inpatient wards have completely incorporated this HIS into their daily lives. This nursing module was adopted using a comparative evaluation as it encompasses the largest group of users and is the most prominent clinical module in any HIS.

### Evaluation methods

In the present study, the usability evaluation of the nursing module of Shafa HIS was performed in the laboratory of health information technology at Kashan University of Medical Sciences using the HE and CW evaluation methods. The results of these expert-based evaluation methods were then compared.

### Heuristic evaluation

Nielsen and Molich first introduced this method [31]. According to the Nielsen approach, a summarized list of heuristic principles is given to evaluators, which can be used as a guideline and then the user interface is independently examined by each evaluator, following which relevant usability problems are recognized [32]. Ten heuristics principles were developed by Nielsen, including visibility of system status, the match between the system and the real world, user control and freedom, consistency and standards, helping users recognize, diagnose, and recover from errors, error prevention, recognition rather than recall, flexibility and efficiency of use, aesthetic and minimalist design, and help and documentation that must be observed in the user interface design [20]. According to this method, evaluators are asked not to share ideas with one another before the evaluation is completed as a single evaluator might fail to recognize a large number of problems, while a variety of evaluators can recognize a wide range of unique issues. Hence, more comprehensive results can be yielded after the findings of a number of HE evaluations are combined [32]. In case of limited time and resources, the usability problems will quickly and economically be identified through this method by three to five evaluators [20, 33, 34].

### Cognitive walkthrough evaluation

The CW is a popular expert-based method with an emphasis on ease of learning the system [22, 35]. This method is considered suitable when users need to masterfully learn a new application or function by learning through exploration. To carry out the CW, the system user interface design needs to be precisely descripted and task scenario, assumptions about the users and the scope of use along with the series of actions that users take to successfully perform a given task need to be determined. Then, a series of cognitive processes followed by users during the performance of a number of actions are simulated by an evaluator or a group of evaluators so as to perform specific tasks. During this phase, the evaluators try to decide on the actions which are not easy for ordinary users by learning the interface behavior and the influence it has on the user. Therefore, this evaluation method could be performed at an early phase of system development to meet user needs [35].

### Participants

As only three to five evaluators are needed to do the HE and CW evaluations [28], the maximum number of evaluators (five) did the evaluation in a random and purposeful fashion. Three evaluators were Ph.D. students in health information management and two evaluators had M.Sc. degrees in health information technology. The evaluators had previous experience with performing the HE and CW evaluations and were familiar with various healthcare information systems [36]. Furthermore, the processes and workflow of the nursing information system were also explained to them.

### Data collection

In this study, the CW evaluation was primarily done by the evaluators in order to prevent the adverse effect of learning the system on the CW evaluation results and, after a period of two weeks, the HE evaluation was performed. Khajouei et al. [28], in order to avoid the effect of learning the system on the results of the HE and CW methods, conducted their study in two phases by reversing the order of evaluators of each method in the first and second phases and after the washing period; no significant difference was observed between the number of recognized problems in the first and second phases of the HE and CW evaluations. Hence, in this study, in order to prevent the effect of learning, the HE evaluation was done two weeks after the CW evaluation method. Nielsen's usability principles were explained to the evaluators and they were told to evaluate the user interface in accordance with the checklist. In order to prevent bias, the HE evaluation was done on those parts of the nursing module in which five CW evaluation scenarios had been performed.

A method suggested by Polson and Lewis was used to do the CW evaluation [22]. Based on nurses’ daily routine tasks and secretaries of inpatient wards, five scenarios were designed using the nursing module based on nurses' opinions and the approval of the head nurse. For each scenario, users' goals and sub-goals, the series of actions performed for each task and the system response were defined. Table 1 shows an example of a scenario including tasks and actions. Each evaluator independently imagined what a real user would do based on background information available to system users and then accomplished the series of actions needed for each task. Following looking at the system from users' point of view, each evaluator determined (a) users’ goals which would lead to actions, (b) if the prompts and labels of the interface would make the user perform the correct task based on the correct goals and (c) the effect of users’ goals on response to the feedback from the interface after performing the action. Any potential issues were reported to the researcher [22]. The comments, questions, and ambiguities raised by the evaluators as well as the problem and its location were recorded by the researcher, who was also considered as the observer. In the final phase of the evaluation process, the evaluators reviewed their problem lists and either added a comment or corrected a previously given comment, if necessary. Then, in a meeting with the researcher and evaluators, all lists were compared and duplicate problems were eliminated and finally a list of individual usability problems was prepared. Finally, this list was provided to the evaluators, who independently determined the level of severity of each problem on a scale ranging from 0 to 4 according to the frequency of the problem, its impact on users, and its persistence [37]. Problem severity was graded as follows:

- 0 = No problem: This is not a usability problem.
- 1 = Cosmetics: Correction is required only if more time is available.
- 2 = Minor: There is a low priority for problem correction.
- 3 = Major: There is a high priority for problem correction.
- 4 = Catastrophic: The product can be used only if the problem is corrected [36–38].

**Table 1:** An example of a scenario and the related tasks and actions

Scenario	Task	Actions
Recording a surgery request for femoral fracture reduction for a patient in the orthopedic ward	Determining a patient	Click on inpatient ward name (The orthopedic ward)
		Icons and patients' names are displayed
		Right-click on the particular patients' icon
		The drop-down menu is displayed next to the patients' icon
	Entering a request for a surgical procedure	Choose “send to operating room waiting list” from the drop-down menu
		A list of available operating rooms is displayed
		Click on “general operating room”
		The general operating room window is displayed
		The patients' surgery information is added
	Submitting a surgery request to the operating room	Click on the Save button
		A message titled “Information was saved successfully” is displayed
		Click on the Confirm button
		“Patients in the surgery waiting list” is displayed
		Click on the Return button
		Inpatient ward window is displayed

The ISO and Nielsen usability attributes were used to recognize usability problems [12, 13]. Table 2 shows the usability attributes of both the ISO and Nielsen. Evaluators independently assigned the recognized problems to one of the usability attributes.

**Table 2:** The usability attributes according to the ISO and Nielsen [12, 13]

Attribute	Definition
Effectiveness	How well do the users reach the goals set with the usage of the system?
Efficiency	How much of each resource (e.g., time and mental effort) is required so that the goals can be obtained by users?
Satisfaction	How pleasant is the use of the system for users?
Learnability	How easy is it for users to do basic tasks when using the system for the first time?
Memorability	When the system has not been in use for a while, how easy can users remember how to use it?
Errors	When using the system, how many errors are made, how severe are these errors and how easily can they be retrieved?

Following a two-week period, once the CW evaluation was completed, five evaluators were asked to independently evaluate the user interface of the nursing module using the Nielsen's usability principles checklist, which was based on the Xerox heuristics checklist [39]. This checklist had been designed according to Nielsen's ten usability principles and included 254 items with multiple-choice questions where options included "Yes," "No," and "Not Applicable" answers. The validity and reliability of the checklist had previously been confirmed in a study by Rezaei-Hachesu et al. [40]. The recognized problems were then listed in the problem report form, which comprised a four-column table including "problem title," "problem description," "location of the problem," and "violated usability principle" as the head of columns.

Subsequently, the recognized problems were analyzed in a meeting with the five evaluators, where the repeated problems were discarded and a comprehensive list of unique problems was made. Moreover, disagreements about the identified problems were resolved in this meeting. Finally, similar to the CW evaluation, the evaluators independently determined the level of severity of each problem on a scale of 0–4 and assigned the recognized problems to a specific usability attribute.

### Data analysis

The data was analyzed using SPSS Statistics for Windows, version 20.0 (SPSS Inc., Chicago, Ill., USA) using both descriptive and inferential statistical techniques. The average severity of the usability problems was calculated. Then, each usability problem was assigned into one of the five categories shown in Table 3, according to its average severity [41]. Furthermore, each problem was associated with one of the usability attributes in which it had the most frequency. If the problem was classified into more than one usability attribute based on its maximum frequency, the problem was associated with the most relevant usability attribute according to the evaluators' opinion [36].

**Table 3:** Problem categories according to their average severity [41]

Problem category	Average severity
No problem	0–0.5
Cosmetic	0.6–1.5
Minor	1.6–2.5
Major	2.6–3.5
Catastrophic	3.6–4

The one-variable Chi-square test was used to compare the total number of recognized usability problems and the number of recognized problems in terms of usability attributes by the two methods. Moreover, the Chi-square test was used to compare the number of recognized problems according to the usability attributes in each of the two evaluation methods. The average severity levels of the problems identified in both methods were compared using the Mann–Whitney U test. A significance level of 0.05 was considered in this study.

## Results

The present study compared the usability evaluation results obtained by the HE and CW methods in the nursing module of an HIS. The HE method was able to identify 317 problems, of which 104 unique problems remained after merging the problems and discarding the repeated ones. Similarly, 46 problems were recognized in the CW evaluation, of which 24 unique problems remained after merging the problems and discarding the repeated ones; the one-variable Chi-square test indicated a significant difference between the number of the recognized problems in the two evaluation methods (*P* < 0.001).

In this study, 57 problems (54.8%) were considered as minor problems and 33 of them (31.7%) were considered as major problems out of the unique problems recognized in the HE method. In the CW evaluation, 12 problems (50%) were considered as major problems and 10 problems (41.7%) were considered as minor problems out of the unique problems recognized. The average severity of usability problems was 2.34 and 2.77 in the HE and CW methods, respectively, and the Mann–Whitney U test revealed a significant difference based on these figures (Table 4).

**Table 4:** Number and severity of the recognized problems by the two evaluation methods

Methods	Number of problems		Number of unique problems based on severity				Mean ± SD	Mann–Whitney test
	Total	Unique	Cosmetic N (%)	Minor N (%)	Major N (%)	Catastrophic N (%)		
HE	317	104	8 (7.7)	57 (54.8)	33 (31.7)	6 (5.8)	2.34 ± 0.65	Z = –3.082 *P* = 0.002
CW	46	24	–	10 (41.7)	12 (50)	2 (8.3)	2.77 ± 0.51	

**Fig. 1:**
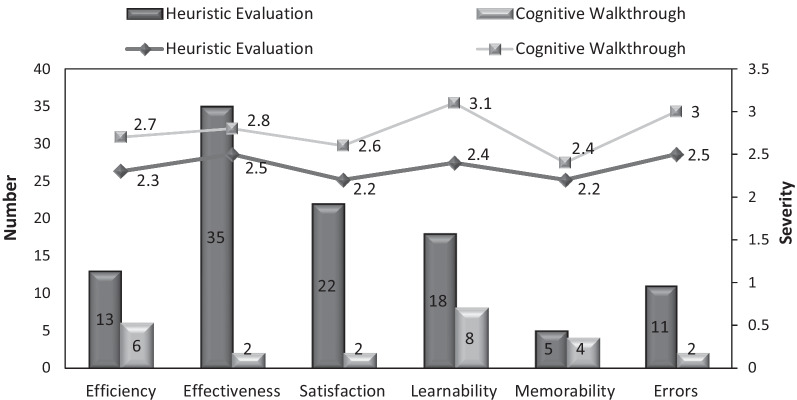
Comparison of the number and severity of problems in the HE and CW methods according to the usability attributes

The number and average severity of the recognized problems according to the usability attributes are shown in Fig. 1.

According to Chi-square test, the difference between the number of the recognized problems based on usability attribute in the HE method was significant (*P* < 0.001). Most of the problems recognized through the HE method were related to effectiveness and satisfaction. In the CW method, while most recognized problems were related to learnability and efficiency, no significant difference was observed between the number of the recognized problems based on usability attributes in this method (*P* = 0.156).

According to Table 5, a significant difference was observed between the total number of the identified problems in the HE and CW evaluation methods (*P* < 0.001). The HE method identified more problems in all usability attributes than the CW method and there was a significant difference between the two methods based on efficacy, effectiveness, satisfaction, learnability and error (*P* < 0.001). However, the number of recognized problems in terms of memorability did not show a statistically significant difference (*P* > 0.05).

**Table 5:** Comparison of the two methods in recognizing problems according to usability attributes

Usability methods	HE N (%)	CW N (%)	Chi-square tests	Only HE N (%)	Only CW N (%)	Both HE and CW N (%)
*Usability attributes*						
Efficiency	13 (12.5)	6 (25)	0.0108	12 (12.5)	5 (31.2)	1 (12.5)
Effectiveness	35 (33.7)	2 (8.3)	< 0.001	34 (35.4)	1 (6.2)	1 (12.5)
Satisfaction	22 (21.2)	2 (8.3)	< 0.001	22 (23)	2 (12.5)	0 (0)
Learnability	18 (17.3)	8 (33.3)	0.05	15 (15.6)	5 (31.2)	3 (37.5)
Memorability	5 (4.8)	4 (16.7)	0.739	3 (3.1)	2 (12.5)	2 (25)
Errors	11 (10.6)	2 (8.3)	0.013	10 (10.4)	1 (6.2)	1 (12.5)
Total	104 (100)	24 (100)	< 0.001			
Percentage of the problems recognized by the two methods to the total number of problems				96 (80)	16 (13.3)	8 (6.7)

From among the total number of recognized problems in two methods (n = 128), 80% were identified by the HE method, 13.3% by the CW method and 6.7% by both methods. In other words, 33.3% (n = 8) of the recognized problems in the CW method overlapped with the recognized problems in the HE method. Regarding efficiency, effectiveness and error, one problem and for learnability and memorability, three and two problems were identified by both methods.

## Discussion

The usability evaluation of the nursing module of Shafa HIS was performed by the HE and CW evaluation methods. The higher number of problems was recognized by the HE method as compared to the CW method, while the average severity of recognized usability problems by the HE was less than that of the CW method.

A significant difference was observed between the total number of usability problems recognized by the HE and CW methods in the usability evaluation of the nursing module. In the studies conducted by Jeffries et al. [26] and Frøkjær and Lárusdóttir [25], it was also indicated that the HE method can recognize more usability problems compared to the CW evaluation, which is compatible with the results of the present study. It should be noted that the number of recognized problems in the HE method depends on the expertise and skill of the evaluators [26, 42–45]. In another study performed by Khajouei et al. [28], it was indicated that no significant difference was seen between the numbers of recognized problems in these two evaluation methods, which was incompatible with the findings of the present study. This may be because Nielsen's usability principles checklist was used in the present study, while in the study conducted by Khajouei et al. [28], no usability checklist was used to carry out the HE method. As a way to confirm this notion, another study done by Khajouei et al. [34] showed that using a checklist in performing the HE method can lead to identifying a higher number of usability problems than when the evaluation is performed without a checklist.

Based on the obtained results, more than half of the recognized problems by the HE method were considered as minor problems, while %50 of the problems recognized by the CW evaluation were considered as major problems and the average severity of usability problems in this method was significantly higher than that of the HE method. Some studies indicated that most of the recognized problems by the HE method are in the minor or cosmetic categories [14, 44, 46], which is compatible with the findings of the present study. Contrary to these findings, other studies reported that most of the recognized problems by the HE method were major and catastrophic [47, 48]. In a study performed by Jeffries et al. [26], it was revealed that two-thirds of the problems identified by the HE method were low-severity problems. The difference in the types of recognized problems due to investigating different health information systems can be considered as a source of this inconsistency. The skill and expertise of evaluators, which is highly effective in estimating the severity of recognized problems [43], can be another reason for the inconsistency observed in findings.

The comparison of the average severity between the two methods based on the usability attribute showed that the average severity of learnability problems in the CW method was significantly higher than that of the HE method. Cuomo and Bowen [24] proposed that the CW evaluation was the superior method in identifying certain problems that either affect user performance or focus on how users interact with the user interface while performing specific tasks [49]. Therefore, as a task-independent method, the HE method illustrates various aspects of a system user interface and identifies specific problems that users hold in low regard for taking corrective actions as compared with the CW method.

The HE method recognized more problems in each of the usability attributes than the CW method, leading to a statistically significant difference between the number of recognized problems in both methods based on all usability attributes except memorability. In the study conducted by Khajouei et al. [28], the number of satisfaction problems identified in the HE method was significantly higher than that of the CW method, which is consistent with the results of the present study. However, they also did not observe a significant difference in the number of problems among other usability attributes, which is not consistent with the results of the present study. As previously mentioned, Nielsen’s usability principles checklist was used in the study done by of Khajouei et al. [28], therefore more problems in each of the usability attributes were observed in the HE method compared to the CW method in this study, which was not unexpected. However, we have no assumptions about the generalizability of this result in the evaluation of other information systems, and a similar study on other information systems might yield different results.

In another study done by Khajouei et al. [16], the HE method identified a significantly higher number of problems related to satisfaction, learnability and error attributes than the think aloud method as one of the usability testing methods, which is consistent with the results of the present study. Usability testing is a widely used technique to evaluate system utility in achieving goals from the perspective of end users [50] and is used to assess the ease of system use and identify the problems that users encounter when working with the system [51]. Nielsen considers usability testing to be the most appropriate method due to end-user participation. However, he suggests expert-based evaluation methods in order to gather additional usability information [19]. According to a study conducted by Paz et al. [52], many usability problems identified through usability testing had previously been identified through the HE evaluation; however, there were significant differences regarding the significance of the problems identified through both methods. Therefore, it would be desirable to perform user-based and expert-based evaluation methods in order to validate the results of each method.

Comparison of the two methods in terms of the overlap between the recognized problems showed that only one-third of the recognized problems in the CW method overlapped with the recognized problems through the HE method. In fact, neither of the two methods alone can identify all usability problems, and the HE method, while identifying more problems in each of the usability attributes, cannot replace the CW evaluation method.

Based on the results of the HE evaluation method, a significant difference was observed between the number of recognized problems based on usability attributes. Most of the attribute problems in this method were related to the effectiveness and satisfaction. In the study done by Khajouei et al. [28], the highest number of recognized problems in the HE method was related to satisfaction and the lowest number of problems was related to effectiveness, and there was no significant difference between the number of problems regarding different attributes. It is acknowledged that health care is a complex environment for information systems due to complex contextual dynamics and rapid changes in its operating context [53, 54]. Furthermore, integration of healthcare information systems into work processes affects their use by healthcare workers [55]. Implementing HISs in such an environment is more complex and costly than implementing other information systems [56]. Successful implementation of an HIS depends on user satisfaction. Therefore, factors influencing user satisfaction should be considered when designing, developing or adopting such systems [57]. Moreover, user satisfaction is often used as a measurement of users’ perception of the effectiveness of an information system [58]. As a rule of thumb, effectiveness can be achieved when users perform their activities and tasks wholly and accurately via the system [59]. In a complex healthcare environment, the effectiveness of health information systems reduces errors by improving legibility, completeness and accuracy of information required for users to make decisions and perform their tasks, having a positive effect on improving quality of care [60–62]. It can be stated that, according to the results of the present study and Khajouei et al.'s study [28], the HE method is a validated method to identify satisfaction problems. Also, according to the results of this study, the HE method is powerful for identifying the problems of effectiveness, which is contrary to the findings reported by Khajouei et al. [28]. Therefore, further studies of this type on different systems are needed to confirm this finding and determine the capabilities of each method to identify other attributes.

According to the results, while in the CW method, the highest number of recognized problems was related to learnability and efficiency, no significant difference was observed between the number of recognized problems based on the usability attribute in this method. In the study done by Khajouei et al. [28], the highest number of problems identified through the CW evaluation method was related to learnability. Learnability refers to the speed at which new or novice users learn a system user interface to perform various tasks [63]. Efficiency refers to the users' time and mental effort and the costs involved to achieve their goals with minimum resources and costs [28, 64]. Within the dynamic healthcare environment, healthcare information systems are being constantly implemented, changed and updated [65–67]. Meanwhile, usability problems and users’ unfamiliarity with the user interface lead users to spend more time completing tasks by the system, hence, decreasing the efficiency [59, 62]. On the other hand, training time in HISs is highly limited for the healthcare workers such as nurses who are essentially required to learn how to work with the system while doing their duties [68]. By performing the CW evaluation and modifying the usability problems recognized by this method can ultimately decrease user cognitive load and system learning time and increase system efficiency.

### Strengths and weaknesses

This study compared two expert-based usability evaluation methods in a nursing module as the most important and widely used HIS module. Moreover, five evaluators participated in this study, which is an adequate number of evaluators to perform the HE and CW evaluations. Furthermore, the evaluators had previous experience with conducting several HE and CW evaluations and either had work experience or were familiar with various HISs and, according to previous studies [42–44], the participation of skilled and experienced evaluators is beneficial in identifying the most significant number and the most severe usability problems. Another strength of this study was that five scenarios were identified based on nurses' most important and frequent daily tasks performed via the nursing module. These scenarios were formed according to the opinion of three experienced nurses working in the inpatient ward, which the head nurse then approved.

However, evaluating the nursing module of the HIS can be considered as a limitation in this study. Since designing different user interfaces for various HISs can influence the number and severity of recognized problems and consequently the number of problems attributed to each usability attribute in each of the HE and CW methods, the findings of this study cannot be fully generalized to all HISs. Another limitation of this study is the possibility of the effect of CW evaluation on the results of the HE evaluation method, while, according to Khajouei et al.'s findings [28], performing the CW before the HE has no effect on its results. However, in this study, the number of problems identified in the HE method was more than that of the CW evaluation method.

## Conclusion

Comparing the HE and CW methods regarding the usability evaluation of a nursing module, it seems that the HE method can identify a higher number of problems compared to the CW evaluation method due to its broad overview of a system design based on a set of predefined principles, while the evaluation of specific tasks in detail is avoided. However, users may be less concerned with adopting corrective measures for these problems. Additionally, this study indicated that performing a reasonable number of tasks in the CW evaluation leads to identifying fewer problems with higher average severity than the HE method. Also, according to the results of this study, the HE evaluation seems to prove more effective and accurate in identifying usability problems affecting effectiveness and satisfaction and the CW method seems to be more suitable for identifying learnability problems with higher average severity.

## Supplementary Information

**Additional file 1**. Study data extraction sheet.

## Data Availability

The required data to analyze and achieve the purpose of this study are presented in this published article and its Additional file 1.

## Abbreviations

HIS: Hospital Information System
HE: Heuristic Evaluation
CW: Cognitive Walkthrough
ISO: International Organization for Standardization
